# Mir-22 inhibits the proliferation, migration, and invasion of human CD133-positive glioblastoma stem cells

**DOI:** 10.55730/1300-0152.2781

**Published:** 2025-11-05

**Authors:** Sevil KÖSE, Özlenen ÖZKAN, Ömer ÖZKAN

**Affiliations:** Department of Plastic, Reconstructive and Aesthetic Surgery, Faculty of Medicine, Akdeniz University, Antalya, Turkiye

**Keywords:** Glioblastoma, glioma, microRNA, miR-22, cancer stem cell, CD133

## Abstract

**Background/aim:**

Glioblastoma multiforme (GBM) is one of the most aggressive and fatal malignancies of the central nervous system. Despite advancements in treatment strategies, effective therapies for GBM remain insufficient, necessitating further improvements. Notably, miR-22 has been found to be significantly downregulated in both glioblastoma tissues and cell lines. In this study, we aim to evaluate miR-22 expression levels in GBM (U87) and CD133-positive (CD133^+^) GBM stem cells (GSCs) and to investigate its effects on proliferation, colony formation, migration, invasion, and wound-healing in U87 and CD133^+^ U87 cells in vitro.

**Materials and methods:**

We isolated CD133^+^ U87 cells using magnetic-activated cell sorting and determined the percentage of CD133^+^ cells by flow cytometry. qRT-PCR detected miR-22 expression. We transfected miR-22 miRNA into U87, CD133^+^, and CD133^−^ U87 cells using a lipid-based transfection reagent. Cell viability was assessed spectrophotometrically on days 1, 3, 5, and 7 using the CCK-8 viability assay. Transwell assays were used to analyze migration and invasion. Wound healing was assessed using a scratch assay.

**Results:**

MiR-22 expression was lower in CD133^+^ U87 cells than in U87 cells. MiR-22 overexpression suppressed proliferation in U87, CD133^+^, and CD133^−^ U87 cells. MiR-22 overexpression also inhibited migration and invasion in both CD133^+^ and CD133^−^ U87 cells and impaired wound-healing capacity in both U87 and CD133^−^ U87 cells.

**Conclusion:**

These results suggest that miR-22 acts as a tumor suppressor in GBM and CD133^+^ GSCs. Therefore, miR-22 represents a potential therapeutic target for cancer stem cell-based glioblastoma treatment.

## Graphical abstract

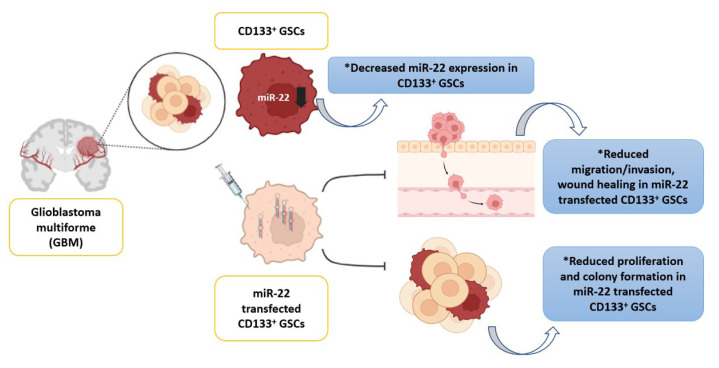

## 1. Introduction

Glioblastoma multiforme (GBM) is the most common and aggressive primary brain tumor in adults, accounting for approximately 45% of all primary brain tumors and 55% of all gliomas. The annual incidence of GBM is estimated to be three to four cases per 100,000 people in the United States and Europe, with an increasing prevalence in older populations (Wen and Kesari, 2008). Despite advancements in treatment modalities, including surgery, radiation therapy, and chemotherapy, GBM remains a highly lethal disease with a median overall survival of less than 14 months and a 5-year survival rate of less than 6% (Marenco-Hillembrand et al., 2020). The poor prognosis of GBM is primarily attributed to its highly invasive nature, resistance to conventional therapies, and extensive tumor heterogeneity (Jain, 2018; Tan et al., 2020). Cancer stem cells (CSCs) have been identified as key contributors to tumor initiation, progression, therapy resistance, and recurrence (Chu et al., 2024). Furthermore, CD133^+^ stem cells from brain tumors have been shown to possess strong tumor-initiating capabilities (Zeppernick et al., 2008). Analysis of GBM tumor tissues from 95 cases revealed that CD133 expression correlates with GBM patient survival (Brescia et al., 2013) and is essential for maintaining glioblastoma stem cell (GSC) stemness (Zeppernick et al., 2008).

MicroRNAs (miRNAs) are small, noncoding RNAs that regulate gene expression at the posttranscriptional level, influencing various cellular processes, including proliferation, differentiation, and apoptosis (O’Brien et al., 2018). It is estimated that miRNAs regulate approximately 60% of human genes, with 70% of known miRNAs being expressed in the brain (Kou et al., 2020). MiR-22, an evolutionarily conserved miRNA, is widely expressed in various tissues, including the brain (Cao et al., 2020; Hu et al., 2020a; Wang et al., 2020), where it has been detected in both neurons and glial cells (Jimenez-Mateos et al., 2015). Recent studies have demonstrated that miR-22-3p (referred to as miR-22) is significantly downregulated in human GBM tissues and cell lines (Chen et al., 2016; Hu et al., 2020b; Ma et al., 2021; Rastegar-Moghaddam et al., 2022; Tu et al., 2022). Mechanistically, miR-22 has been shown to suppress tumor cell proliferation, migration, and invasion (Chen et al., 2016; Ma et al., 2021), while promoting apoptosis and cell cycle arrest in the G2/M phase (Zhang et al., 2020a). Furthermore, miR-22 overexpression enhances glioma cell sensitivity to cisplatin, suggesting its potential to improve therapeutic efficacy (Zhang et al., 2020a; Rastegar-Moghaddam et al., 2022). However, its role in GSC tumorigenesis remains unclear.

In this study, we propose that miR-22 acts as a tumor suppressor in GBM and CD133^+^ GSCs, underscoring its potential as a therapeutic target. We examined miR-22 expression in CD133^+^ GSCs and investigated its impact on key malignant phenotypes, including proliferation, colony formation, migration, invasion, wound healing, and temozolomide (TMZ) resistance. To our knowledge, this is the first report elucidating the regulatory role of miR-22 in these oncogenic processes in GSCs.

## 2. Materials and methods

### 2.1. Study design

**Figure 1:**
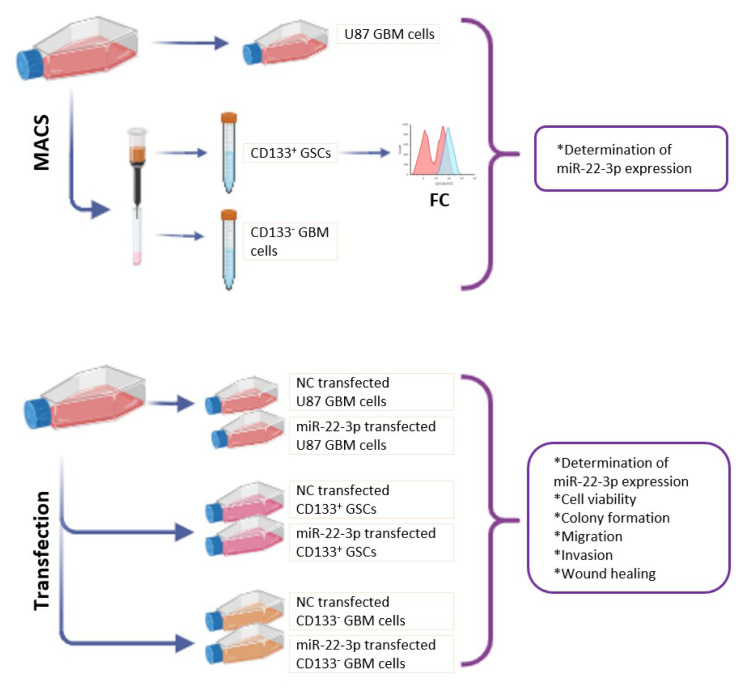
Schematic design of the study.

We designed a prospective, randomized, controlled in vitro study. Independent variables are time points (1, 3, 5 and 7 days) and groups [(TMZ) treated and untreated U87, CD133^+^ U87 and CD133^−^ U87 cells; miR-22-3p (i.e. miR-22)-transfected and miRNA mimic negative control (NC)-transfected negative control U87, CD133^+^ U87 and CD133^−^ U87 cells], dependent variables include quantitative measurements of miRNA expression, CD133^+^ labeling, cellular metabolic activity, colony formation, migration, invasion and wound healing. In this in vitro study, we used a commercially available U87 cell line and did not involve any human or animal subjects. Therefore, ethics committee approval was not required. However, all experimental procedures were conducted in accordance with ethical standards and guidelines to ensure the integrity and ethical compliance of the research. Biological replicates were determined by power analysis (G*Power v3.1) using an effect size of 0.8, a power (one–β) of 0.8, and α = 0.05. Figure 1 illustrates the experimental setup schematically.

### 2.2. Cell culture

We purchased the U87 MG (U87, #HTB-14) cell line from the American Type Culture Collection (ATCC, USA). Cells were cultured in DMEM (#41965039; Gibco, USA) containing 10% of FBS (#P30-193306; PAN-Biotech, Germany) and incubated at 37 °C with 5% CO_2_ (Onay et al., 2022). CD133^+^ GSCs were cultured in a serum-free DMEM/F12 medium (#11320033; Gibco) supplemented with 10 ng/mL basic fibroblast growth factor (#100-18B; Gibco), 20 mg/mL epidermal growth factor (#AF-100-15; Gibco), and 1% NCS21 neuronal supplement (#C21-H; Capricon) under 5% CO_2_ at 37 °C (Liu et al., 2018).

### 2.3. Magnetic-activated cell sorting (MACS)

CD133^+^ U87 cells were isolated using MACS with the human CD133 MicroBead Kit (#130-097-049; Miltenyi Biotec, USA). Cultured cells were rinsed with PBE buffer (PBS with 0.5% BSA (#9418; Sigma-Aldrich, USA) and 2 mM EDTA (#E6511; Sigma-Aldrich) and incubated with a CD133 antibody-labeled magnetic bead suspension at 4 °C for 30 min. The cell suspension was then placed in a separation column (#130-042-401; Miltenyi Biotec) within a magnetic field, and CD133^−^ cells were collected. After removing the separation column from the magnetic field, the CD133^+^ cells were collected (Kose et al., 2018; Korkusuz et al., 2019).

### 2.4. Flow cytometry (FC)

The permeabilized cells (Permeabilizing Solution 2, #340973; BD Biosciences) were indirectly immunolabeled using FITC Mouse Anti-Human CD133 antibody (#567029; BD Pharmingen). Labeled cells were analyzed using a FACS Aria III (Becton Dickensen) and evaluated with FACSDiva software (Becton Dickensen) with 1000 events (Kose et al., 2018; Kose et al., 2024a).

### 2.5. RNA isolation and qRT–PCR

For miRNA expression analysis, RNA was isolated using the EcoPURE Total RNA kit (#E2075; Ecotech Biotech, Türkiye) according to the manufacturer’s instructions, and reverse-transcribed into cDNA using the miRNA All-In-One cDNA Synthesis Kit (#G898; ABM, Canada). The cDNA was then used to measure miRNA expression and assess transfection efficiency via qRT–PCR using A.B.T. 2X qPCR SYBR-Green MasterMix (#Q03-02-05, Türkiye). qRT–PCR reactions were performed with CFX96 real-time System (Bio-Rad, USA), and expression profiles were evaluated using the 2^−ΔΔCT^ method (Kose et al., 2018). U6 snRNA (MS00033740) was used as an internal control for miRNA. All sequences of primers were listed: U6, forward: 5′-GCTTCGGCAGCACATATACTAAAAT-3′, reverse: 5′-CGCTTCACGAATTTGCGTGTCAT-3′; miR-22, forward: 5′-GTTCTTCAGTGGCAAGC-3′, reverse: 5′-GAACATGTCTGCGTATCTC-3′.

### 2.6. Transfection

Cells were seeded at a density of 20 × 10^4^ cells/well, and transfection was performed using Lipofectamin Transfection Reagent (#18324012; Invitrogen, USA) with hsa-miR-22 miRNA Mimic (#MCH01594; ABM) or miRNA Mimic Negative Control (#MCH00000; ABM) for each well. Cells were harvested using a cell scraper in ice-cold PBS 48 h after transfection for RNA isolation (Zhang et al., 2020a).

### 2.7. Cell viability assay

Cell viability was assessed using the Cell Counting Kit-8 (CCK8, #KTA1020; Abbkine, China), following the manufacturer’s instructions. MiR-22-transfected U87, CD133^+^, and CD133^−^ cells with or without 212,3 μM TMZ (Kocak Farma, Temomid, Türkiye) were seeded in 96-well plates at a density of 1 × 10^4^ cells/well (Bisht et al., 2024). After culturing for 1, 3, 5, and 7 days, 10 μL of CCK-8 solution was added into each well, and the cells were incubated for another 4 h. Absorbance was measured at 450 nm (Multiscan go; Thermo Scientific, USA) (Kose et al., 2024a).

### 2.8. Colony formation assay

Cells were seeded in 24-well plates at 1 × 10^2^ cells/well in 0.8% agar (#A7921; Sigma) in growth medium over a 1% agar layer. The plates were incubated for 20 days until colonies were large enough to be visualized. To detect colony numbers, colonies were stained by 0.2% crystal violet. Colonies were visualized using a microscope, and photographs were taken and counted using the Axiocam imaging system and the Zen 3.3 program (Liu et al., 2018).

### 2.9. Transwell migration and invasion assay

The migration assay was performed in 0.5-μm 24-well transwells (#3422; Corning, USA). For the invasion assay, filters coated with Matrigel (#CLS356255; Corning). Briefly, 5 × 10^4^ cells were seeded into the upper chamber with 100 μL of serum-free medium, and 600 μL of growth medium was added to the lower chamber. After 48 h of incubation, cells in the upper chamber were removed using cold PBS and fixed with 3.7% paraformaldehyde. The chamber was stained with 0.2% crystal violet (#109218; Merck, Germany) for 30 min at room temperature. Migrated cells were visualized using a microscope (Zeiss, Primovert, Germany), and photographs were taken using the Axiocam imaging system (Zeiss) and the Zen 3.3 program (Zeiss). Five area measurements were performed from each of the three filters using the ImageJ program (USA). To determine the number of migrated cells, filters were incubated in 0.25% trypsin EDTA solution (Cytiva, #SH30042, USA) for 5 min. The cells were then counted using a Thoma slide after labeling with 0.4% tripan blue (#15250061; Gibco) (Kose et al., 2018; Kose et al., 2024b).

### 2.10. Wound healing

Cells were cultured in 24-well plates (one × 10^5^ cells/well). When the cell monolayer reached confluence, scratch wounds were created with a 200-μL micropipette tip and washed with PBS. The cells were maintained under standard conditions. The wound area was visualized under a microscope at 10× magnification at 0 and 24 h, and photographs were taken. The wound area was measured using the Zen 3.3 program. The closure of the wound area was calculated as the ratio between the relative residual wound area at the beginning and at the end of the assay (Chen et al., 2016).

### 2.11. Statistical analysis

Data are presented as the means ± SD and analyzed using GraphPad Prism (GraphPad Software). One-way ANOVA and two-way ANOVA with Tukey’s multiple comparisons test (for comparisons between multiple groups) were used. A p-value of < 0.05 was considered statistically significant.

## 3. Results

### 3.1. miR-22 expression is lower in CD133 positive glioblastoma cells

**Figure 2:**
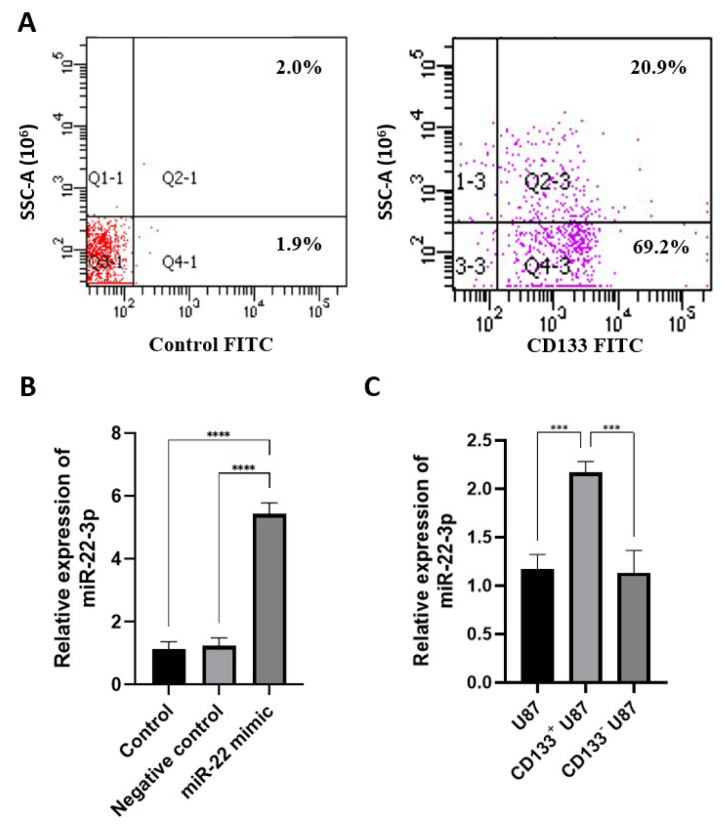
qRT–PCR analysis revealing that miR-22 expression is higher in CD133^+^ U87 than in U87 and CD133^−^ U87 cells; (A) CD133 labeling of the cells isolated from U87 cells using MACS; (B) qRT–PCR analysis of miR-22 expression in control, NC and miR-22-transfected U87 cells; (C) qRT–PCR analysis the expression levels of miR-22 in U87, CD133^+^ and CD133^−^ U87 cells (n = 3) (* p < 0.05, ****p < 0.0001).

As a result of CD133 isolation from 1 × 10^7^ U87 cells, 1.2 × 10^4^ CD133^+^ U87 cells were obtained. It was determined that 90.1% of the cells isolated from U87 cells using MACS were CD133 positive (Figure 2A). We found that miR-22 expression was upregulated in miR-22 mimic-transfected U87 cells compared with untransfected U87 cells and miRNA mimic NC-transfected U87 cells (p < 0.0001) (Figure 2B). Also, miR-22 expression was found to be lower in CD133^+^ U87 cells compared with U87 and CD133^−^ U87 cells (p < 0.05) (Figure 2C).

### 3.2. miR-22 overexpression suppresses glioblastoma and GSC proliferation

**Figure 3:**
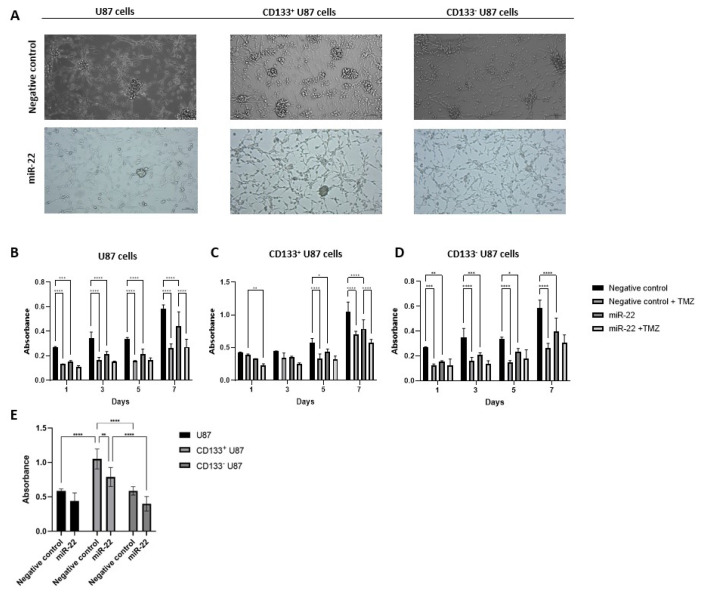
MiR-22 overexpression suppress CD133 positive glioblastoma cell proliferation; (A) microscopic analysis of the morphology of U87, CD133^+^ U87 and CD133^−^ U87 cells after transfected NC and miR-22 miRNA (10×); viability analysis in NC and miR-22-overexpressed (B) U87, (C) CD133^+^ U87 and (D) CD133^−^ U87 cells; (E) viability analysis in U87, CD133^+^ U87 and CD133^−^ U87 cells transfected with NC and miR-22 on day 7 (n = 3) (*p < 0.05, **p < 0.01, ***p < 0.001, ****p < 0.0001).

Phase contrast microscopy images revealed that spheroid formation increased in CD133^+^ U87 cells compared with U87 and CD133^−^ U87 cells (Figure 3A). Additionally, spheroid formation decreased in all U87, CD133^+,^ and CD133^−^ U87 cell groups following miR-22 overexpression (Figure 3A). Cell growth was significantly reduced in miR-22-transfected U87 cells compared with NC cells at each time point (p < 0.05) (Figure 3B). TMZ inhibited cell growth in NC U87 at each time point (p < 0.001) (Figure 3B). Furthermore, TMZ significantly inhibited cell growth in miR-22-overexpressing U87 cells on day 7 (p < 0.0001) (Figure 3B). In miR-22-transfected CD133^+^ U87 cells, cell growth was significantly reduced compared with NC cells on days 5 and 7 (p < 0.05 and p < 0.0001, respectively) (Figure 3C). TMZ inhibited cell growth in NC CD133^+^ U87 cells on days 5 and 7 (p < 0.0001) (Figure 3C). TMZ significantly inhibited cell growth in miR-22 overexpressing CD133^+^ U87 cells on day 7 (p < 0.0001) (Figure 3C). Cell growth was significantly reduced in miR-22-transfected CD133^−^ U87 cells compared with NC cells at each time point (p < 0.05) (Figure 3D). TMZ inhibited cell growth in NC CD133^−^ U87 cells at each time point (p < 0.0001) (Figure 3D). When the viability analyses were compared between NC and miR-22-transfected U87, CD133^+^ and CD133^−^ U87 cells on day 7, NC CD133^+^ U87 cell growth was found to be significantly higher than that of NC U87 and NC CD133^−^ U87 cells (p < 0.0001) (Figure 3E). Additionally, miR-22-transfected CD133^+^ U87 cell growth was significantly higher than that of miR-22-transfected CD133^−^ U87 cells (p < 0.0001) (Figure 3E).

### 3.3. miR-22 impairs colony formation capacity in U87, CD133 positive and negative glioblastoma cells

**Figure 4:**
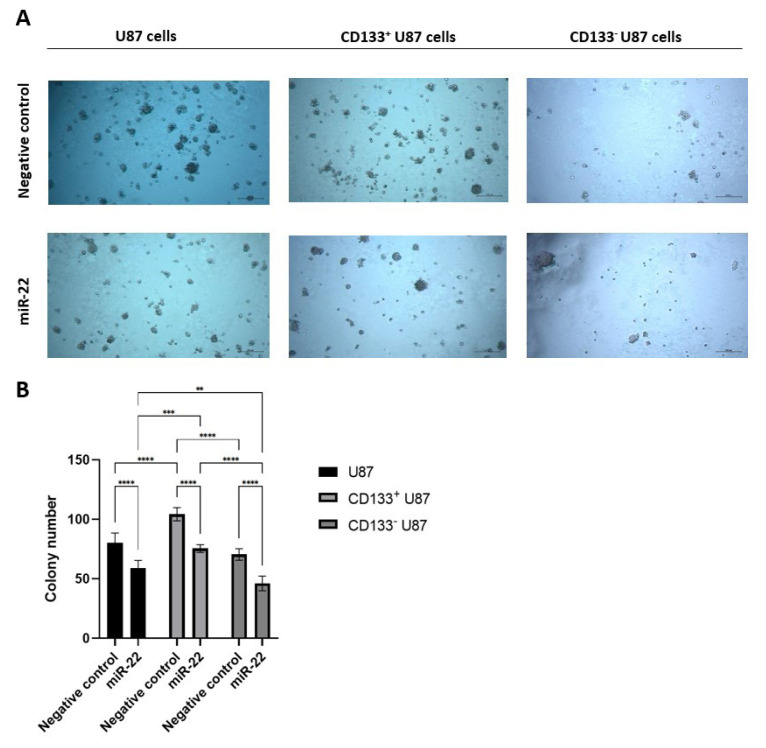
MiR-22 suppresses colony formation ability in both U87, CD133^+^ and CD133^−^ U87 cells; (A) morphologic analysis of the NC and miR-22-transfected U87, CD133^+^ U87 and CD133^−^ U87 cells (10×); (B) colony number of NC and miR-22-transfected U87, CD133^+^ U87 and CD133^−^ U87 cells on day 10 (n = 3) (**p < 0.01, ***p < 0.001, ****p < 0.0001).

Microscopic analysis and colony quantification demonstrated that miR-22 overexpression significantly reduced colony formation capacity in all cell groups compared with their NC cells (p < 0.0001) (Figure 4A, Figure 4B). MiR-22 transfection led to a significant decrease in colony numbers across all cell groups (U87, CD133^+^ and CD133^−^ U87 cells) relative to their NC controls (p < 0.0001) (Figure 4B). The colony number was higher in NC CD133^+^ U87 cells compared with NC U87 and NC CD133^−^ U87 cells (p < 0.0001) (Figure 4B). Additionally, colony numbers were lower in miR-22-transfected CD133^−^ U87 cells compared with miR-22-transfected U87 and CD133^+^ U87 cells (p < 0.01 and p < 0.0001, respectively) (Figure 4B). Furthermore, miR-22-transfected U87 cells compared with miR-22-transfected CD133^+^ U87 cells (p < 0.001) (Figure 4B).

### 3.4. miR-22 suppresses migration and invasion in both CD133 positive and negative glioblastoma cells

**Figure 5:**
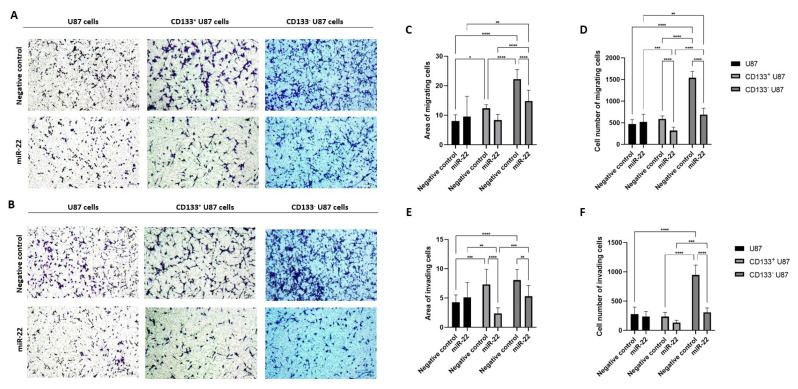
MiR-22 suppresses migration and invasion in both CD133^+^ and CD133^−^ U87 cells; morphologic analysis of the migrated (A) and invaded (B) NC and miR-22-overexpressed U87, CD133^+^ U87 and CD133^−^ U87 cells (10×); area of the migrated (C) and invaded (E) NC and miR-22-overexpressed U87, CD133^+^ U87 and CD133^−^ U87 cells; cell number of migrated (D) and invaded (F) NC and miR-22-overexpressed U87, CD133^+^ U87 and CD133^−^ U87 cells (n = 3) (*p < 0.05, **p < 0.01, ***p < 0.001, ****p < 0.0001)

In all cell groups, miR-22 overexpression significantly reduced the number of migrating (Figure 5A) and invading (Figure 5B) cells compared with the NC cells. Migration was decreased in CD133^−^ U87 cells compared with their NC cells, as determined by cell number measurements (p < 0.0001) (Figure 5C). Additionally, migration in NC CD133^+^ and CD133^−^ U87 cells was significantly higher than in NC U87 cells (p < 0.05) (Figure 5C). However, no significant difference was observed between miR-22 overexpressing U87 cells and their NC cells (Figure 5C). MiR-22 overexpression also led to a greater reduction in migration in CD133^+^ and CD133^−^ U87 cells compared with miR-22 overexpressing U87 cells (p < 0.05) (Figure 5C, Figure 5D). Migration was decreased in CD133^−^ U87 cells compared with their NC cells, as determined by area measurements (p < 0.0001) (Figure 5D). Similarly, CD133^+^ U87 cell migration was reduced compared with NC cells, according to cell count measurements (p < 0.0001) (Figure 5D). No significant difference was observed between miR-22 overexpressing U87 cells and their NC cells (Figure 5D). Regarding invasion, miR-22 overexpression led to a significant decrease in invasion in CD133^+^ U87 cells compared with miR-22 overexpressing U87 cells, as indicated by the invading cell area measurements (p < 0.01) (Figure 5E). CD133^+^ U87 cell invasion decreased compared with NC cells, according to the invading cell area measurement results (p < 0.0001) (Figure 5E). However, no significant difference was found between miR-22 overexpressing U87 cells and NC cells (Figure 5E, Figure 5F). Furthermore, NC CD133^+^ and CD133^−^ U87 cells exhibited higher invasion rates than NC U87 cells (p < 0.05) (Figure 5E, Figure 5F). CD133^−^ U87 cells exhibited significantly lower invasion compared with their NC cells, based on both cell count and area measurement (p < 0.01 and p < 0.0001, respectively) (Figure 5E, Figure 5F).

### 3.5. miR-22 impairs wound-healing capacity in both U87 and CD133 negative glioblastoma cells

**Figure 6:**
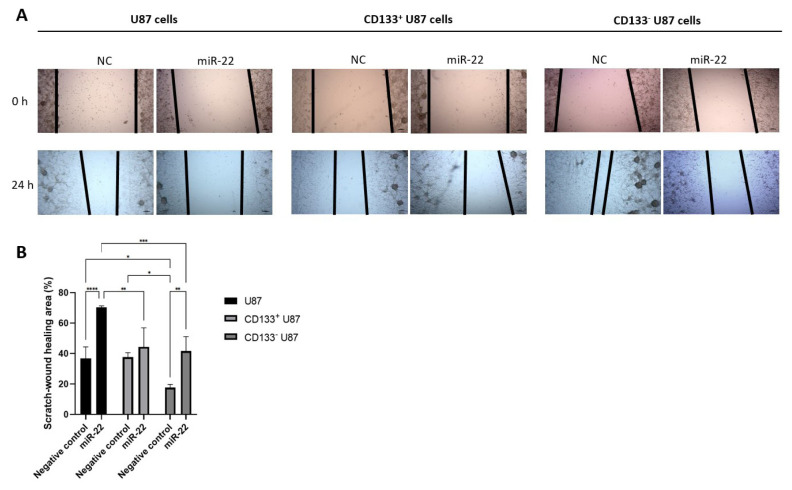
MiR-22 suppresses wound-healing ability in both U87 and CD133^−^ U87 cells; (A) morphologic analysis of the migrated NC and miR-22-transfected U87, CD133^+^ U87 and CD133^−^ U87 cells (10×); (B) the rate of wound closure in NC and miR-22-overexpressed U87, CD133^+^ U87 and CD133^−^ U87 cells at 24 h (n = 3) (*p < 0.05, **p < 0.01, ***p < 0.001, ****p < 0.0001).

Microscopic analysis revealed that miR-22 overexpression significantly reduced wound closure ability in all cell groups compared with the NC cells (Figure 6A). The wound closure rate was significantly lower in miR-22-transfected U87 and CD133^−^ U87 cells compared with their respective NC cells (p < 0.0001 and p < 0.01, respectively) (Figure 6B). NC CD133^−^ U87 cells exhibited a higher wound closure rate than NC U87 and NC CD133^+^ U87 cells (p < 0.05) (Figure 6B). Additionally, miR-22 overexpressing U87 cells displayed a lower wound closure rate compared with miR-22 overexpressing CD133^+^ and CD133^−^ U87 cells (p < 0.01 and p < 0.001, respectively) (Figure 6B).

## 4. Discussion

We determined that 86.2% of cells isolated from U87 cells using MACS were CD133-positive. Other studies reported that 88.1% and 85% of CD133^+^ cells were isolated from U87 cells using the same method (Liu et al., 2018; Pavon et al., 2012). These findings suggest that MACS is a reliable method for isolating CD133^+^ cells from U87 cells, yielding consistent results across studies. Additionally, we found that the U87 cell line contained 1.2% CD133^+^ cells. In studies where separation was performed using magnetic beads, the CD133^+^ cell proportion in U87 cells was reported as 1.4% (Liu et al., 2018). Other studies using FC to isolate CD133^+^ cells from U87 cells have shown that CD133^+^ cell rates of 1.1% (Jung et al., 2013), 0.5% (Ping et al., 2007), 2.4% (Lee et al., 2020), 2.1% (Wei et al., 2022) and 1.6% (Zhang et al., 2017). Overall, the obtained results are generally comparable; however, the differences observed between studies using the FC isolation method may be attributed to factors such as antibody specificity and experimental conditions (Pavon et al., 2012).

Our results indicate that, following miR-22 mimic transfection, miR-22 expression in U87 cells increased fivefold compared with both untransfected and NC-transfected U87 cells. In other studies using the lipid-mediated transfection method, miR-22 expression in U87 cells increased fourfold (Han et al., 2020) and fivefold (Zhang et al., 2020a) compared with untransfected cells. The transfection duration was not specified in the study (Han et al., 2020); however, based on the protocol, we selected a 48-h transfection period over the 24-h option and determined that transfection efficiency was sufficient. In the plasmid vector-mediated transfection, miR-22 expression was increased sevenfold in human GBM cell lines U251 (Ma et al., 2021) and LN18 (Ma et al., 2021); fivefold and eightfold in human GBM cell lines DAOY (Xu et al., 2014) and ONS-76 (Xu et al., 2014), respectively, compared with untransfected and NC-transfected cells, as assessed by qRT–PCR. Although we used a liposomal-based transient transfection method in our study, we achieved expression efficiency comparable to that of other methods. We found that miR-22 expression was lower in CD133^+^ U87 cells than in U87 and CD133^−^ U87 cells. While no previous study has specifically examined miR-22 expression in CD133^+^ GSCs or GBM tissue, a study by Zekri et al. reported that miR-22 expression was reduced in CD133^+^ primary hepatocellular carcinoma cells compared with primary hepatocellular carcinoma cells (Zekri et al., 2018). Our findings provide convincing evidence that reduced miR-22 expression in CD133^+^ GCSs is significantly associated with malignant features, including metastasis, recurrence, and resistance to chemotherapy and radiotherapy. These findings suggest that miR-22 may serve as a novel prognostic marker for this disease (Wang et al., 2013; Rastegar-Moghaddam et al., 2022).

In our study, as in previous reports (Sun et al., 2012; Jung et al., 2013; Kanno et al., 2013), spheroid formation was morphologically evaluated and found to be increased in CD133^+^ U87 cells compared with U87 and CD133^−^ U87 cells. Our results indicate that cell proliferation decreased following miR-22 overexpression in U87, CD133^+^, and CD33^−^ U87 cells compared with their NC controls. Consistently, previous studies have reported decreases in cell growth on days 3 (Chen et al., 2016) and 4 (Han et al., 2020; Zhang et al., 2020a) in miR-22-transfected U87 cells compared with NC U87 cells, as assessed by CCK-8 and WST-1 assays, respectively. Similarly, in our study, we observed that miR-22-transfected U87 cells exhibited reduced proliferation compared with NC controls on days 3 and 5. In contrast, a study using primary human GBM cells reported no significant difference in cell proliferation between miR-22-transfected and NC-transfected cells, as assessed by CCK-8 on day 2 (Tu et al., 2022). In our study, a difference in proliferation was observed between U87 and miR-22-overexpressed U87 cells on day 1. This discrepancy may be attributed to differences in cell types or variations in transfection duration, which were not specified in the study (Tu et al., 2022), despite the use of similar transfection and analysis methods. Nevertheless, we found that cell growth was significantly reduced in miR-22 overexpressing CD133^+^ U87 cells compared with NC CD133^+^ U87 cells on day 7. Notably, several studies have investigated the effects of miR-22 transfection on U87 glioblastoma cell viability; however, to the best of our knowledge, no studies have evaluated these effects in CD133^+^ U87 GSCs. Therefore, our study provides the first analysis of miR-22’s impact on the viability of CD133^+^ U87 cells.

In our study, we found that 212.3 μM TMZ significantly reduced U87 and CD133^−^ U87 cell proliferation at all time points, while it reduced CD133^+^ U87 cell proliferation on days 5 and 7. XTT analysis revealed that 250 μM TMZ reduced cell proliferation in CD133^+^ U87 cells, obtained from U87 cells cultured in neural stem cell medium, compared with untreated cells on day 1 (Behrooz et al., 2022). In our study, however, there was no difference in the viability of CD133^+^ U87 cells treated with TMZ compared with those not treated with TMZ on day 1. This lack of difference may be attributed to factors such as the TMZ dose, variations in CD133^+^ cell yield, or differences in the method used to assess viability. Another study found that 400 μM TMZ applied for 1 day or 250 μM TMZ applied for 3 days to CD133^+^ U87 cells (isolated from U87 cells using the MACS method) reduced cell proliferation, as measured by XTT analysis, compared with untreated cells (Liu et al., 2018). In our study, TMZ inhibited cell growth in NC CD133^+^ U87 cells on days 5 and 7. We used the TMZ dose that provided 50% lethality in U87 cells (Bisht et al., 2024). The difference in TMZ dose between studies may have contributed to the extended effect duration in our study (Bisht et al., 2024). Additionally, in our study, we found that miR-22 overexpression in U87 or CD133^+^ U87 cells did not affect TMZ sensitivity, which, to our knowledge, is reported for the first time in the literature.

This study highlights that miR-22 overexpression suppresses migration and invasion in both CD133^+^ U87 and CD133^−^ U87 cells. There was no difference in migration and invasion between miR-22-overexpressed cells and NC cells in U87 cells. Previous studies have shown that miR-22 overexpression suppresses migration and invasion in GBM cell lines U251 and LN18 (Ma et al., 2021), and invasion in U87 cells (Chen et al., 2016; Zhang et al., 2020a) compared with NC cells, as assessed by the transwell invasion assay. Although there was no difference in migration and invasion between miR-22-overexpressed and NC cells in U87 cells, miR-22 suppresses migration and invasion in both CD133^+^ and CD133^−^ U87 cells. There is no information in the literature regarding the changes in migration and invasion ability after miR-22 transfection of CD133^+^ U87 cells, and this is analyzed for the first time in this study. When examining the wound-healing properties of cells, we found that miR-22 impaired wound-healing capacity in both U87 and CD133^−^ U87 cells. The wound-healing assay revealed that miR-22 decreased wound-healing capacity in U87 cells, from 600 pixels to 300 pixels, compared with NC cells (Chen et al., 2016). This percentage difference in recovery parallels the percentage difference between miR-22-transfected U87 cells and NC cells.

This study shows that miR-22 overexpression impaired colony-forming capacity in U87, CD133^+^, and CD133^−^ U87 cells. Colony formation capacity decreased threefold in primer GBM cells (Han et al., 2020), twofold in GBM cell lines U251 (Ma et al., 2021), LN18 (Ma et al., 2021), and GSC8-11 (Zhang et al., 2020b) following miR-22 transfection, compared with their NC controls. In our study, consistent with these findings, the colony numbers in miR-22-transfected cells were 1.5 times higher than those in NC controls. It has also been reported that, independent of miR-22 expression, the colony number of CD133^+^ U87 cells was twofold higher than the colony number of U87 and CD133^−^ U87 cells (Liu et al., 2018). Similarly, we determined that the colony number of CD133^+^ U87 cells was 1.5-fold higher than that of U87 and CD133^−^ U87 cells.

To further understand the molecular mechanisms underlying these phenotypic effects, we explored potential miR-22-3p targets using multiple in silico prediction tools (TargetScan, miRDB, miRWalk, and miRanda). By intersecting the predicted target lists, we identified a core set of genes common to at least three algorithms. Gene Ontology and Kyoto Encyclopedia of Genes and Genomes enrichment analyses of these shared targets revealed significant associations with pathways regulating cell proliferation, migration, and invasion—including PI3K-Akt, MAPK, and Wnt signaling pathways. Among the predicted targets, SFRP2 and PCDH15 emerged as the most relevant candidates. Given that SFRP2 activates Wnt/β-catenin signaling and PCDH15 contributes to cellular adhesion and motility, their suppression by miR-22 could mechanistically account for the reduced proliferation, migration, and invasion observed in our study.

In conclusion, the present study demonstrates that miR-22-3p expression is lower in CD133^+^ GSCs and that miR-22 overexpression directly inhibits the viability, colony formation, migration, invasion, and wound healing of CD133^+^ GSCs. These preclinical results are based on an in vitro monolayer cell culture model using a single GBM cell line, U87. The findings can be further tested with different GBM cell lines and in vivo in GBM animal models. Nevertheless, the results highlight, for the first time, the essential role of miR-22 in regulating the stemness and chemoresistance of U87 GCSs. MiR-22-3p functions as a tumor suppressor in GBM, offering new perspectives on GSC-based anti-glioblastoma therapies.

## Data Availability

No datasets were generated or analyzed during the current study.

## References

[b1-tjb-49-07-800] Behrooz AB Vazifehmand R Tajudin AA Masarudin MJ Sekawi Z 2022 Tailoring drug co-delivery nanosystem for mitigating U-87 stem cells drug resistance Drug Delivery and Translational Research 12 1253 1269 10.1007/s13346-021-01017-1 34405338

[b2-tjb-49-07-800] Bisht P Prasad SR Choudhary K Pandey R Aishwarya D 2024 Naringin and temozolomide combination suppressed the growth of glioblastoma cells by promoting cell apoptosis: network pharmacology, in-vitro assays and metabolomics based study Frontiers in Pharmacology 15 1431085 10.3389/fphar.2024.1431085 39148542 PMC11325085

[b3-tjb-49-07-800] Brescia P Ortensi B Fornasari L Levi D Broggi G 2013 CD133 is essential for glioblastoma stem cell maintenance Stem Cells 31 857 869 10.1002/stem.1317 23307586

[b4-tjb-49-07-800] Cao Y Liu H Zhang J Dong Y 2020 Circular RNA cZNF292 silence alleviates OGD/R-induced injury through up-regulation of miR-22 in rat neural stem cells (NSCs) Artificial Cells, Nanomedicine, and Biotechnology 48 594 601 10.1080/21691401.2020.1725536 32052645

[b5-tjb-49-07-800] Chen H Lu Q Fei X Shen L Jiang D 2016 miR-22 inhibits the proliferation, motility, and invasion of human glioblastoma cells by directly targeting SIRT1 Tumor Biology 37 6761 6768 10.1007/s13277-015-4575-8 26662303

[b6-tjb-49-07-800] Chu X Tian W Ning J Xiao G Zhou Y 2024 Cancer stem cells: advances in knowledge and implications for cancer therapy Signal Transduction and Targeted Therapy 9 170 10.1038/s41392-024-01851-y 38965243 PMC11224386

[b7-tjb-49-07-800] Han M Wang S Fritah S Wang X Zhou W 2020 Interfering with long non-coding RNA MIR22HG processing inhibits glioblastoma progression through suppression of Wnt/beta-catenin signalling Brain 143 512 530 10.1093/brain/awz406 31891366 PMC7009478

[b8-tjb-49-07-800] Hu J Zhou W Zhou Z Yang Q Xu J 2020a miR-22 and cerebral microbleeds in brainstem and deep area are associated with depression one month after ischemic stroke Brazilian Journal of Medical and Biological Research 53 e9162 10.1590/1414-431x20209162 32348425 PMC7197650

[b9-tjb-49-07-800] Hu T Wang F Han G 2020b LncRNA PSMB8-AS1 acts as ceRNA of miR-22-3p to regulate DDIT4 expression in glioblastoma Neuroscience Letters 728 134896 10.1016/j.neulet.2020.134896 32151711

[b10-tjb-49-07-800] Jain KK 2018 A Critical Overview of Targeted Therapies for Glioblastoma Frontiers in Oncology 8 419 10.3389/fonc.2018.00419 30374421 PMC6196260

[b11-tjb-49-07-800] Jimenez-Mateos EM Arribas-Blazquez M Sanz-Rodriguez A Concannon C Olivos-Ore LA 2015 microRNA targeting of the P2X7 purinoceptor opposes a contralateral epileptogenic focus in the hippocampus Scientific Reports 5 17486 10.1038/srep17486 26631939 PMC4668358

[b12-tjb-49-07-800] Jung TY Choi YD Kim YH Lee JJ Kim HS 2013 Immunological characterization of glioblastoma cells for immunotherapy Anticancer Research 33 2525 2533 23749904

[b13-tjb-49-07-800] Kanno H Sato H Yokoyama TA Yoshizumi T Yamada S 2013 The VHL tumor suppressor protein regulates tumorigenicity of U87-derived glioma stem-like cells by inhibiting the JAK/STAT signaling pathway International Journal of Oncology 42 881 886 10.3892/ijo.2013.1773 23338840

[b14-tjb-49-07-800] Korkusuz P Kose S Yersal N Onen S 2019 Magnetic-Based Cell Isolation Technique for the Selection of Stem Cells Methods in Molecular Biology 1879 153 163 10.1007/7651_2018_151 30306535

[b15-tjb-49-07-800] Kose S Aerts-Kaya F Kopru CZ Nemutlu E Kuskonmaz B 2018 Human bone marrow mesenchymal stem cells secrete endocannabinoids that stimulate in vitro hematopoietic stem cell migration effectively comparable to beta-adrenergic stimulation Experimental Hematology 57 30 41 e31 10.1016/j.exphem.2017.09.009 29030083

[b16-tjb-49-07-800] Kose S Onen S Gizer M Boduroglu E Gonullu U 2024a Cannabinoid receptor ligands modulate fibrosis and inflammation in idiopathic pulmonary fibrosis: a preliminary study Turkish Journal of Biology 48 379 389 10.55730/1300-0152.2713 39758842 PMC11698192

[b17-tjb-49-07-800] Kose S Varan C Onen S Nemutlu E Bilensoy E 2024b 2-AG-loaded and bone marrow-targeted PCL nanoparticles as nanoplatforms for hematopoietic cell line mobilization Stem Cell Research & Therapy 15 341 10.1186/s13287-024-03902-1 39354544 PMC11446023

[b18-tjb-49-07-800] Kou X Chen D Chen N 2020 The Regulation of microRNAs in Alzheimer’s Disease Frontiers in Neurology 11 288 10.3389/fneur.2020.00288 32362867 PMC7180504

[b19-tjb-49-07-800] Lee CAA Banerjee P Wilson BJ Wu S Guo Q 2020 Targeting the ABC transporter ABCB5 sensitizes glioblastoma to temozolomide-induced apoptosis through a cell-cycle checkpoint regulation mechanism Journal of Biological Chemistry 295 7774 7788 10.1074/jbc.RA120.013778 32317280 PMC7261782

[b20-tjb-49-07-800] Liu N Hu G Wang H Li Z Guo Z 2018 PLK1 inhibitor facilitates the suppressing effect of temozolomide on human brain glioma stem cells Journal of Cellular and Molecular Medicine 22 5300 5310 10.1111/jcmm.13793 30133120 PMC6201353

[b21-tjb-49-07-800] Ma C Wang H Zong G He J Wang Y 2021 EGR1 modulated LncRNA HNF1A-AS1 drives glioblastoma progression via miR-22-3p/ENO1 axis Cell Death Discovery 7 350 10.1038/s41420-021-00734-3 34772911 PMC8590016

[b22-tjb-49-07-800] Marenco-Hillembrand L Wijesekera O Suarez-Meade P Mampre D Jackson C 2020 Trends in glioblastoma: outcomes over time and type of intervention: a systematic evidence based analysis Journal of Neuro-Oncology 147 297 307 10.1007/s11060-020-03451-6 32157552

[b23-tjb-49-07-800] O’Brien J Hayder H Zayed Y Peng C 2018 Overview of MicroRNA Biogenesis, Mechanisms of Actions, and Circulation Frontiers in Endocrinology (Lausanne) 9 402 10.3389/fendo.2018.00402 PMC6085463 30123182

[b24-tjb-49-07-800] Onay O Kose S Suslu N Korkusuz P Nemutlu E 2022 Human laryngeal squamous cell carcinoma cell line release of endogenous anandamide and 2-arachidonoylglycerol, and their antiproliferative effect via exogenous supplementation: an in vitro study Cell and Tissue Banking 23 93 100 10.1007/s10561-021-09917-9 33797678

[b25-tjb-49-07-800] Pavon LF Marti LC Sibov TT Miyaki LA Malheiros SM 2012 Isolation, cultivation and characterization of CD133+ stem cells from human glioblastoma Einstein (São Paulo) 10 197 202 10.1590/s1679-45082012000200013 23052455

[b26-tjb-49-07-800] Ping YF Yao XH Bian XW Chen JH Zhang R 2007 Activation of CXCR4 in human glioma stem cells promotes tumor angiogenesis Zhonghua Bing Li Xue Za Zhi 36 179 183 17535685

[b27-tjb-49-07-800] Rastegar-Moghaddam SH Ebrahimzadeh-Bideskan A Shahba S Malvandi AM Mohammadipour A 2022 MicroRNA-22: a Novel and Potent Biological Therapeutics in Neurological Disorders Molecular Neurobiology 59 2694 2701 10.1007/s12035-022-02769-8 35156160

[b28-tjb-49-07-800] Sun L Wu Z Shao Y Pu Y Miu W 2012 MicroRNA-34a suppresses cell proliferation and induces apoptosis in U87 glioma stem cells Technology in Cancer Research & Treatment 11 483 490 10.7785/tcrt.2012.500264 22568628

[b29-tjb-49-07-800] Tan AC Ashley DM Lopez GY Malinzak M Friedman HS 2020 Management of glioblastoma: State of the art and future directions CA: A Cancer Journal for Clinicians 70 299 312 10.3322/caac.21613 32478924

[b30-tjb-49-07-800] Tu J Fang Y Han D Tan X Xu Z 2022 MicroRNA-22 represses glioma development via activation of macrophage-mediated innate and adaptive immune responses Oncogene 41 2444 2457 10.1038/s41388-022-02236-7 35279703

[b31-tjb-49-07-800] Wang W Li F Zhang Y Tu Y Yang Q 2013 Reduced expression of miR-22 in gastric cancer is related to clinicopathologic characteristics or patient prognosis Diagnostic Pathology 8 102 10.1186/1746-1596-8-102 23786758 PMC3733645

[b32-tjb-49-07-800] Wang X Shi C Pan H Meng X Ji F 2020 MicroRNA-22 exerts its neuroprotective and angiogenic functions via regulating PI3K/Akt signaling pathway in cerebral ischemia-reperfusion rats Journal of Neural Transmission (Vienna) 127 35 44 10.1007/s00702-019-02124-7 31883035

[b33-tjb-49-07-800] Wei P Jiang J Xiao M Zeng M Liu X 2022 The transcript ENST00000444125 of lncRNA LINC01503 promotes cancer stem cell properties of glioblastoma cells via reducing FBXW1 mediated GLI2 degradation Experimental Cell Research 412 113009 10.1016/j.yexcr.2022.113009 34990616

[b34-tjb-49-07-800] Wen PY Kesari S 2008 Malignant gliomas in adults The New England Journal of Medicine 359 492 507 10.1056/NEJMra0708126 18669428

[b35-tjb-49-07-800] Xu QF Pan YW Li LC Zhou Z Huang QL 2014 MiR-22 is frequently downregulated in medulloblastomas and inhibits cell proliferation via the novel target PAPST1 Brain Pathology 24 568 583 10.1111/bpa.12136 24576181 PMC8029063

[b36-tjb-49-07-800] Zekri AN El-Sisi ER Youssef ASE Kamel MM Nassar A 2018 MicroRNA Signatures for circulating CD133-positive cells in hepatocellular carcinoma with HCV infection PLOS ONE 13e0193709 10.1371/journal.pone.0193709 PMC5849309 29534065

[b37-tjb-49-07-800] Zeppernick F Ahmadi R Campos B Dictus C Helmke BM 2008 Stem cell marker CD133 affects clinical outcome in glioma patients Clinical Cancer Research 14 123 129 10.1158/1078-0432.CCR-07-0932 18172261

[b38-tjb-49-07-800] Zhang G Zhang Y Cheng S Wu Z Liu F 2017 CD133 positive U87 glioblastoma cells-derived exosomal microRNAs in hypoxia- versus normoxia-microenviroment Journal of Neuro-Oncology 135 37 46 10.1007/s11060-017-2566-x 28948499

[b39-tjb-49-07-800] Zhang Y Tu L Zhou X Li B 2020a MicroRNA-22 regulates the proliferation, drug sensitivity and metastasis of human glioma cells by targeting SNAIL1 Journal of BUON 25 491 496 32277674

[b40-tjb-49-07-800] Zhang Z Xu J Chen Z Wang H Xue H 2020b Transfer of MicroRNA via Macrophage-Derived Extracellular Vesicles Promotes Proneural-to-Mesenchymal Transition in Glioma Stem Cells Cancer Immunology Research 8 966 981 10.1158/2326-6066.CIR-19-0759 32350000

